# Improving Prediction of Peroxide Value of Edible Oils Using Regularized Regression Models

**DOI:** 10.3390/molecules26237281

**Published:** 2021-11-30

**Authors:** William E. Gilbraith, J. Chance Carter, Kristl L. Adams, Karl S. Booksh, Joshua M. Ottaway

**Affiliations:** 1 Department of Chemistry, University of Delaware, Newark, DE 19716, USA; wgilbrai@udel.edu (W.E.G.); kbooksh@udel.edu (K.S.B.); 2 Lawrence Livermore National Laboratory, Livermore, CA 94551, USA; carter45@llnl.gov (J.C.C.); adams82@llnl.gov (K.L.A.)

**Keywords:** edible oils, peroxide value, partial least squares regression, ridge regression, LASSO regression, elastic net regression, near infrared, chemometrics, boxcar averaging

## Abstract

We present four unique prediction techniques, combined with multiple data pre-processing methods, utilizing a wide range of both oil types and oil peroxide values (PV) as well as incorporating natural aging for peroxide creation. Samples were PV assayed using a standard starch titration method, AOCS Method Cd 8-53, and used as a verified reference method for PV determination. Near-infrared (NIR) spectra were collected from each sample in two unique optical pathlengths (OPLs), 2 and 24 mm, then fused into a third distinct set. All three sets were used in partial least squares (PLS) regression, ridge regression, LASSO regression, and elastic net regression model calculation. While no individual regression model was established as the best, global models for each regression type and pre-processing method show good agreement between all regression types when performed in their optimal scenarios. Furthermore, small spectral window size boxcar averaging shows prediction accuracy improvements for edible oil PVs. Best-performing models for each regression type are: PLS regression, 25 point boxcar window fused OPL spectral information RMSEP = 2.50; ridge regression, 5 point boxcar window, 24 mm OPL, RMSEP = 2.20; LASSO raw spectral information, 24 mm OPL, RMSEP = 1.80; and elastic net, 10 point boxcar window, 24 mm OPL, RMSEP = 1.91. The results show promising advancements in the development of a full global model for PV determination of edible oils.

## 1. Introduction

The peroxide value (PV) of an edible oil is an indicator of freshness as viewed through oxidative degradation. Chemically, PV is a measurement of the primary oxidation of hydroxyl groups of unsaturated fats in oils by molecular oxygen into hydroperoxides and peroxides [1]. This measurement is often presented in milliequivalents O_2_/kg (mEq O_2_/kg) of oil. Full auto-oxidation of oils further converts the created peroxides and hydroperoxides into alcohols, aldehydes and ketones, which are directly responsible for the rancidity of the oil [2,3]. Fresh oils have peroxide values below approximately 10 mEq O_2_/kg, while oils that have spoiled and became rancid present peroxide values above 30 mEq O_2_/kg [2]. Furthermore, peroxide values as high as 100 mEq O_2_/kg have been linked to cases of food poisoning [4]. The American Oil Chemists Society (AOCS) Official Method Cd 8-53 [5] and the Commission Regulation (EEC) No 2568/91 of 11 July 1991 [6] have established standard iodometric titrations for the determination of edible oil PV in an attempt to maintain product quality control. The titration endpoint, and hence sample PV, is determined by either colorimetric or electrochemical means. However, this established standard method requires toxic chemicals, is labor intensive and time consuming, and requires specialized equipment such as a chemical fume hood; for these reasons, rapid PV analysis in the field is not practical.

Spectroscopy is often the method of choice for real-time, in situ and on-line analysis in the modern age of analytical chemistry. The promise of rapid, non-destructive analysis of simple and complex systems makes spectroscopic measurement methods ideal for production, manufacturing, and quality control applications. Furthermore, in the case of analyzing edible oils, vibrational spectroscopic techniques enable direct determination of PV, removing the need for toxic chemicals and hazardous waste generated from wet chemical PV methods. Infrared spectroscopy-based methods are promising alternatives to wet chemical PV determination. Specifically, Fourier-transform infrared spectroscopy (FTIR) is often cited as a possible replacement for the Official Method Cd 8-53 [7]. FTIR has been shown effective on a variety of individual edible oil types including corn oil [8,9,10], coconut oil [11], palm oil [12], red fruit oil [13], walnut oil [14], vegetable oils [15,16], soybean oil [17,18,19,20], rapeseed/canola oil [17,18,19,20], sunflower oil [8,9,10,17,18,19,20], and olive oil [9,10,18,21,22]. Sample preparation techniques, when combined with FTIR analysis, have been shown to improve prediction models [23]. Furthermore, FTIR has been previously used in thermal aging trials [12,20].

Most published work on PV determination has relied on the prediction of PVs in only a narrowly defined range of edible oil samples and sample degradation conditions. Few publications provide examples of PV determination across a wide range of different edible oil types, oil brands, or an extended PV range [24]. Furthermore, thermal accelerated aging of edible oils, which typically involves high temperatures, often promotes secondary oxidation of the hydroxyl and molecular oxygen species rather than natural primary oxidation; this approach is not optimal for extrapolation of PV studies to real-world applications [25]. Thermal aging also promotes decomposition of the transient hydroperoxide species, further affecting the measured peroxide value [25]. The alternative to accelerated aging is natural oil aging under relevant storage and use conditions. While this latter approach represents the ideal and might lead to more realistic and uniform PV prediction for real-world samples, it requires considerable time, storage space and patience.

Multivariate methods such as partial least squares (PLS) help unlock the hidden potential of spectroscopic analysis to elucidate information hidden within the variance. PLS regression has previously been shown, in many different studies, as an excellent predictor for PVs when combined with infrared (IR) spectroscopy. Specifically, near-infrared (NIR) spectroscopy has been identified as an ideal spectroscopic technique to combine with multivariate analysis for the prediction of PVs of edible oils [7,9,16]. NIR measurements cover the spectral range from 4000 to 12,500 cm^−1^, while FTIR operates in the NIR and mid-infrared (MIR) range from 400 to 4000 cm^−1^. NIR spectroscopy offers an inexpensive and rapid analysis. Few studies have used NIR spectroscopy in a wide variety of different edible oil samples, although examples have shown NIR provides exceptional results in specific cases where limited types of oils were analyzed. Previously, NIR spectroscopy, combined with PLS regression has been shown, in limited variety studies, to predict edible oil PVs within a range of 1.8–17.2 mEq O_2_/kg PV with a root mean squared error of prediction (RMSEP) of 1.87 mEq O_2_/kg PV [9]. However, this same study utilized only three different oil types, namely olive, sunflower and maize oil. Armenta et al. proposes utilizing specific regression models for each edible oil type. However, this design requires knowledge of the sample, as well as updated models for every oil type [9]. 

Recently, Ottaway et al. investigated using multiple vibrational spectroscopy analyses coupled with multivariate methods to both classify naturally aged edible oils by type and to determine the PV [24]. Their studies utilized the linear least squares multivariate methods PLS regression and PLS-discriminant analyses to build the calibration and classification models. Kwofie et al. used a subset of the Ottaway et al. data to perform class differentiation using Raman spectroscopy [26]. Of the 100 samples in 19 different edible oil classes previously shown in Ottaway et al. (Data Set 1), 99 were re-titrated using the AOCS-approved method for peroxide value measurement, resulting in 95 final unique edible oil samples after outlier removal, aged between 3 and 7 years, representing 18 unique oil and oil blend classes, with a range of peroxide values from 5.6 to 80 mEq O_2_/kg.

In this study, a wide variety of naturally aged edible oils were measured using NIR spectroscopy in sample cells having optical pathlengths (OPL) of 2 and 24 mm, respectively. The differing OPLs were required because there was considerable variation in the signal to noise of spectral features measured in the NIR. Spectral regions of lower signal intensity (i.e., lower absorption) benefited from the 24 mm OPL, whereas the shorter 2 mm OPL mitigated saturation of highly absorbing bands. The multivariate analysis methods including partial least squares regression (PLS), ridge regression, LASSO regression and elastic net regression were used to predict the PV. Additionally, the spectral information of the 2 and 24 mm OPL NIR spectra were fused to utilize the most optimal spectral information from each pathlength.

## 2. Results

**Figure A1:**
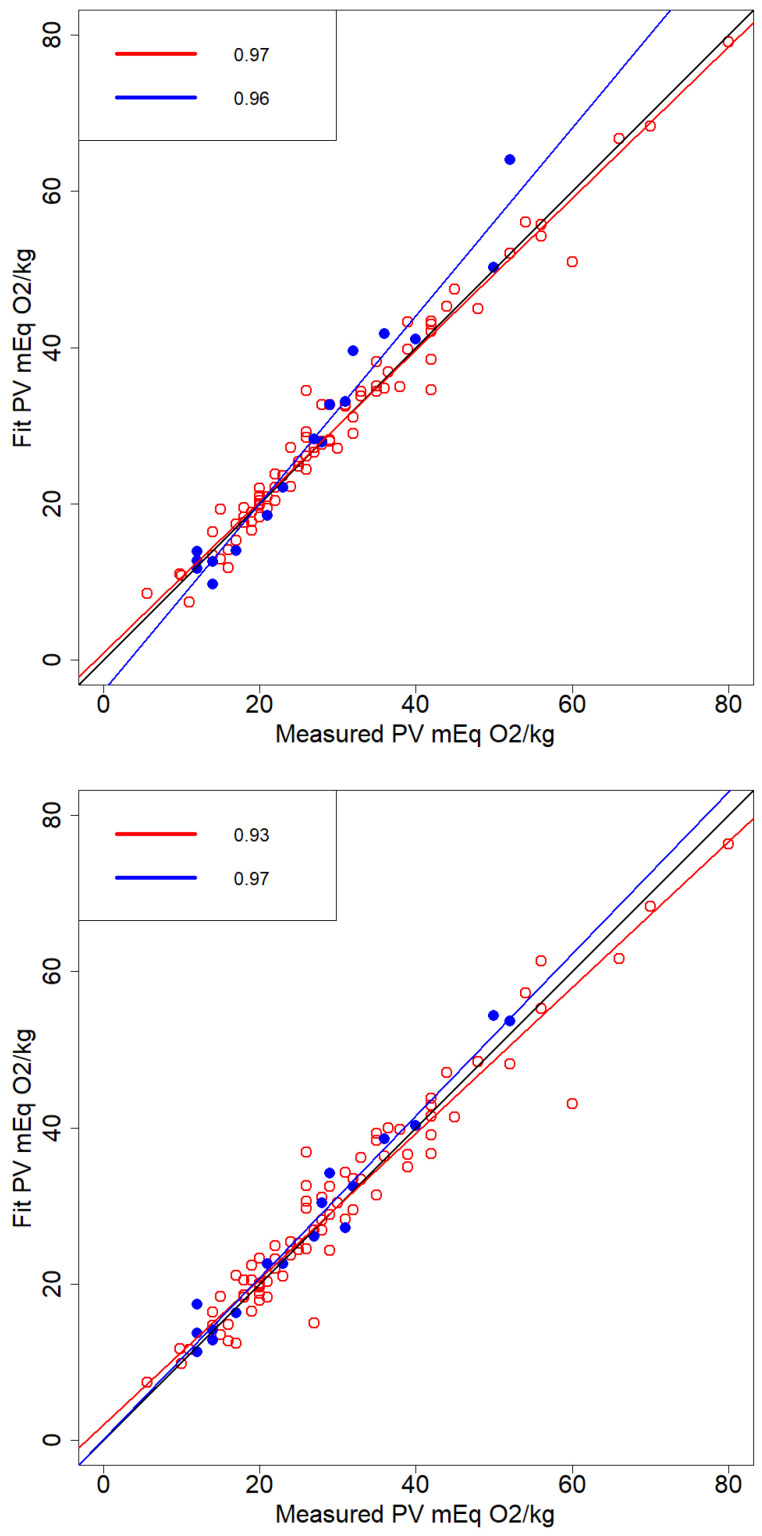

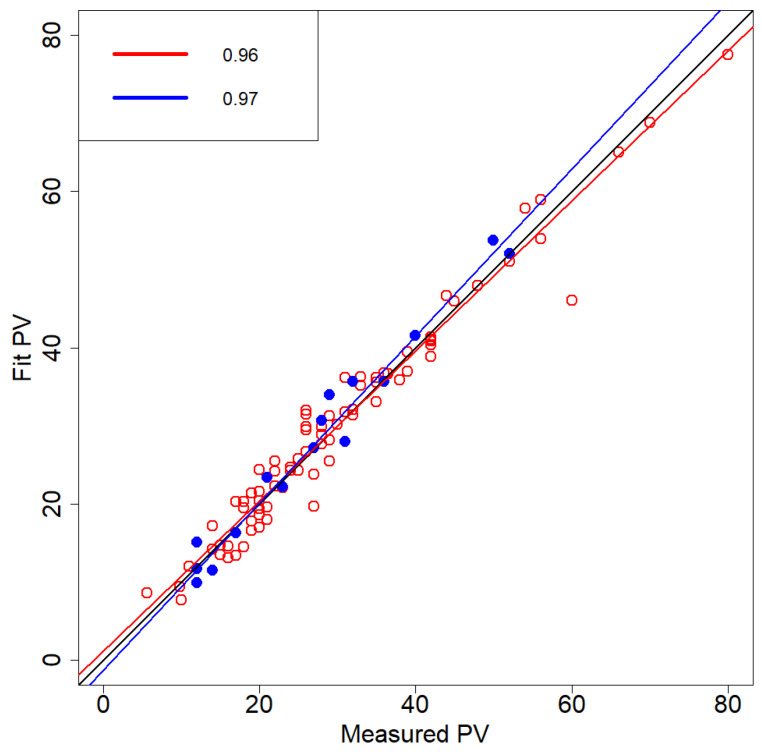
Best-performing PLS prediction regression biplots for each OPL. All figures show calibration set (red points), calibration linear trend (red line), validation set (blue points), validation linear trend (blue line) and line of concordance (black line). The 2 mm OPL, 25 point boxcar window (**top**) RMSEP 4.18 mEq O_2_/kg PV; 24 mm OPL, raw spectra (**middle**) RMSEP 2.59 mEq O_2_/kg PV; fused OPLs, 5 point boxcar window (**bottom**) 2.50 mEq O_2_/kg PV. R^2^ values presented in legend, colored to match biplot linear trends.

**Figure A2:**
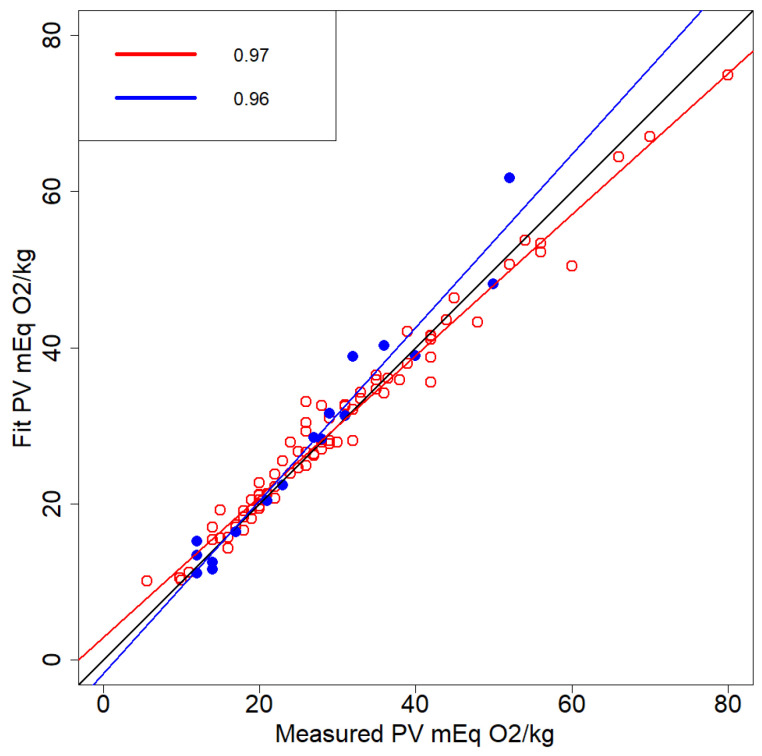

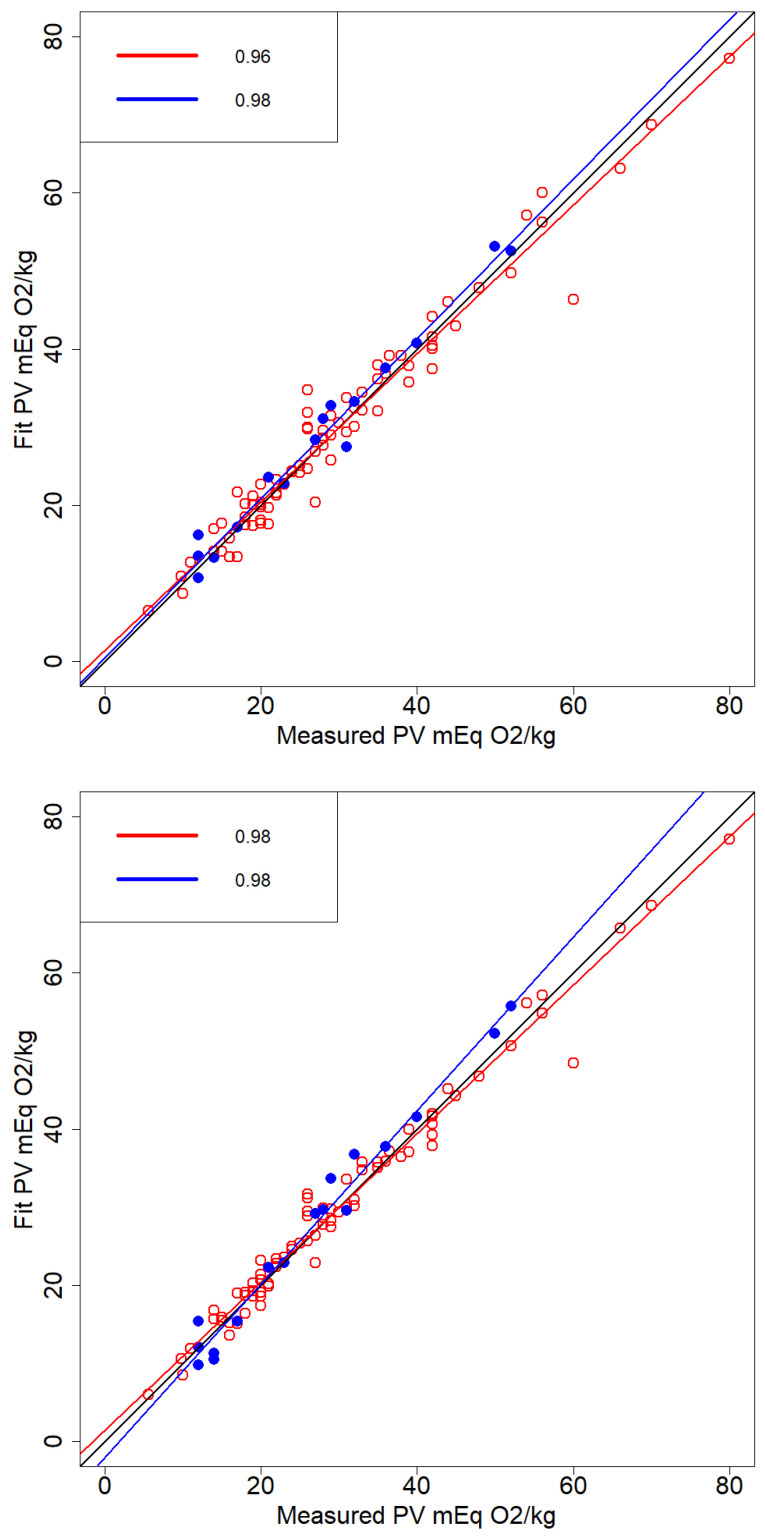
Best-performing ridge prediction regression biplots for each OPL. All figures show calibration set (red points), calibration linear trend (red line), validation set (blue points), validation linear trend (blue line) and line of concordance (black line). The 2 mm OPL, 25 point boxcar window (**top**) RMSEP 3.40 mEq O_2_/kg PV; 24 mm OPL, 5 point boxcar window (**middle**) RMSEP 2.20 mEq O_2_/kg PV; fused OPLs, 25 point boxcar window (**bottom**) 2.66 mEq O_2_/kg PV. R^2^ values presented in legend, colored to match biplot linear trends.

**Figure A3:**
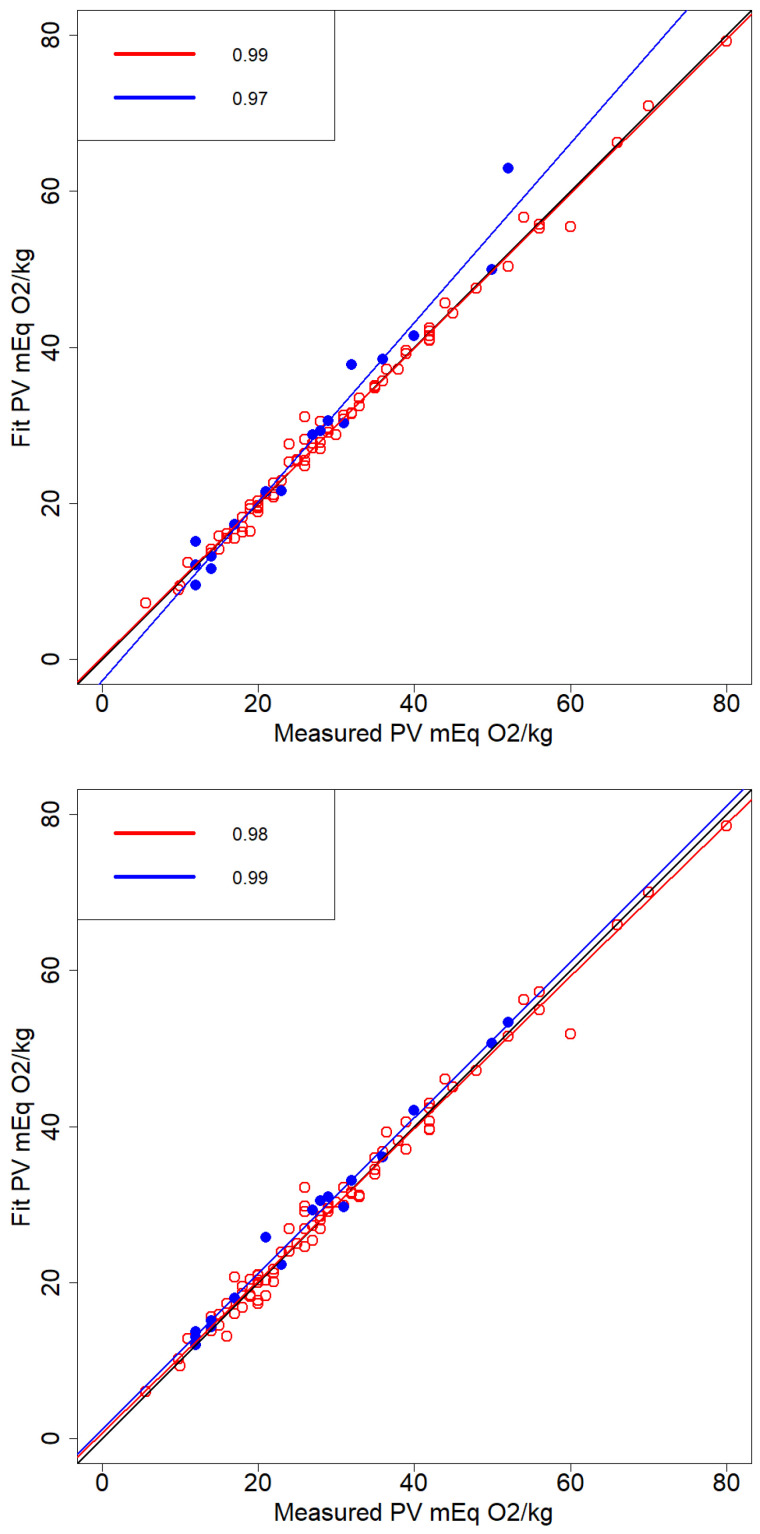

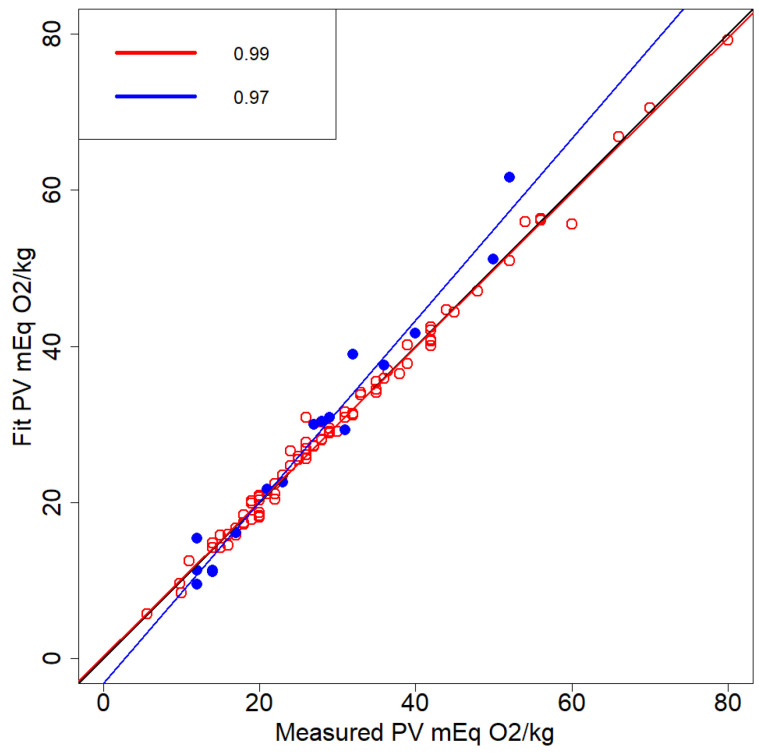
Best-performing LASSO prediction regression biplots for each OPL. All figures show calibration set (red points), calibration linear trend (red line), validation set (blue points), validation linear trend (blue line) and line of concordance (black line). The 2 mm OPL, 25 point boxcar window (**top**) RMSEP 3.39 mEq O_2_/kg PV; 24 mm OPL, raw spectra (**middle**) RMSEP 1.80 mEq O_2_/kg PV; fused OPLs, 25 point boxcar window (**bottom**) 3.48 mEq O_2_/kg PV. R^2^ values presented in legend, colored to match biplot linear trends.

**Figure A4:**
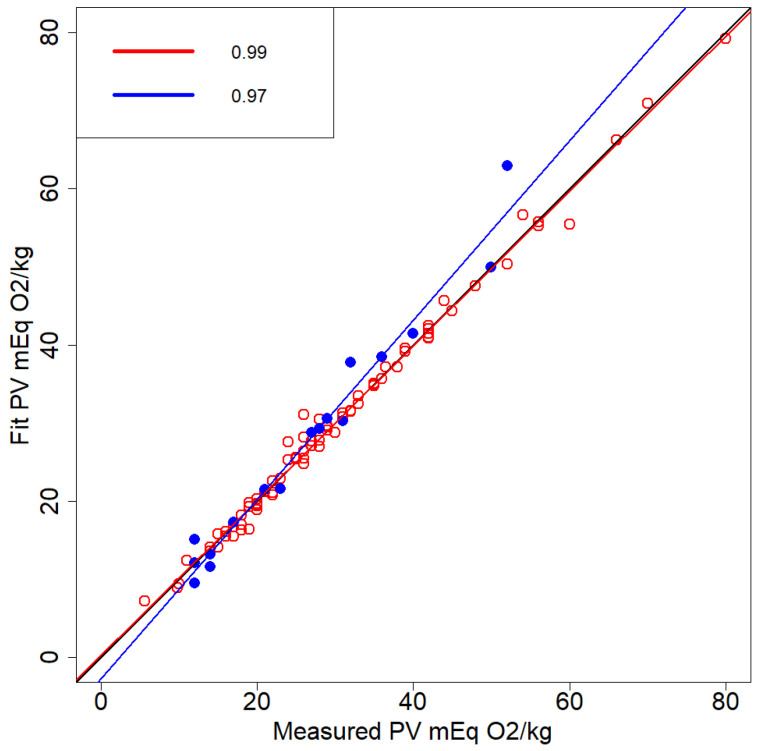

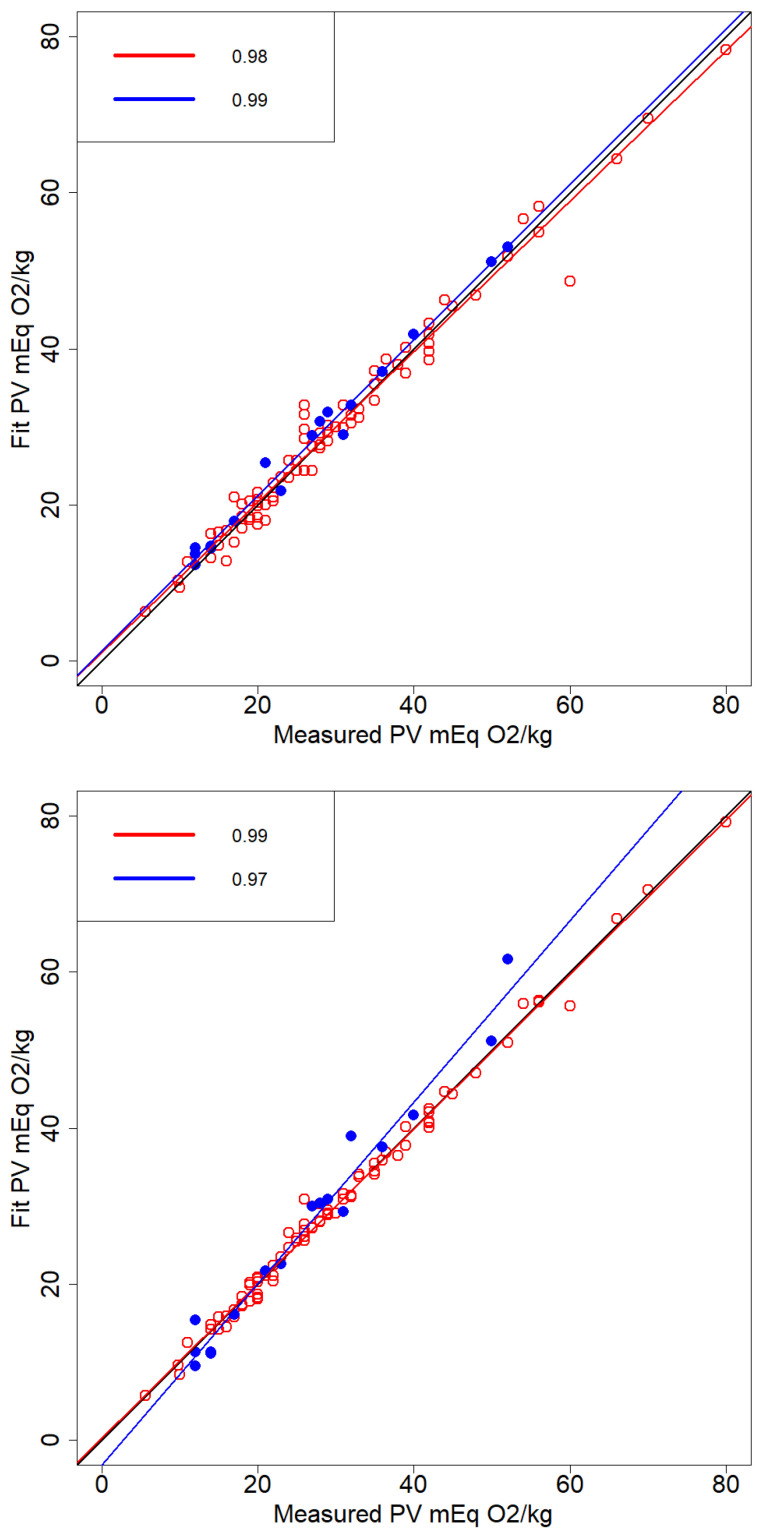
Best-performing elastic net prediction regression biplots for each OPL. All figures show calibration set (red points), calibration linear trend (red line), validation set (blue points), validation linear trend (blue line) and line of concordance (black line). The 2 mm OPL, 25 point boxcar window (**top**) RMSEP 3.39 mEq O_2_/kg PV; 24 mm OPL, 10 point boxcar window (**middle**) RMSEP 1.91 mEq O_2_/kg PV; fused OPLs, 25 point boxcar window (**bottom**) 3.48 mEq O_2_/kg PV. R^2^ values presented in legend, colored to match biplot linear trends.

The PV range of the calibration set was 5.6–80 mEq O_2_/kg PV; the validation set PV range was 12 to 52 mEq O_2_/kg PV. Optimized predictive performance for all methods investigated ranged from 1.80 to 2.50 mEq O_2_/kg PV (Table 1). Models were optimized for degree of boxcar smoothing and OPL of the spectra employed. The best models involved some 1-norm regularization, either LASSO or Elastic Net. The results from each regression technique are discussed in detail below. In all regression techniques, the 2 mm OPL data was shown to be outperformed by the 24 mm OPL data; however, the fused OPL data did marginal improve prediction errors when models were built with PLS. Example regression biplots can be found in Figure A1, Figure A2, Figure A3 and Figure A4 in Appendix A.

### 2.1. PLS

All investigated PLS models, spanning four boxcar averaging windows and three OPL combinations, were found to be optimized with six to nine latent variables (LV) based on RMSECV. The upper end of this LV range is in agreement with the original analyses of this data set by Ottaway [24]. However, fewer LVs were needed for PLS models when more aggressive boxcar averaging was employed. We hypothesize that this trend is due to noise reduction and eliminating chance correlations within lower signal-to-noise (S/N) wavelengths. PLS RMSEP values range from 2.50 to 4.80 mEq O_2_/kg PV (Table 2), showing slight performative increase in certain situations when performed with minimal signal averaging pre-processing steps. The best-performing subset of the PLS regression models utilized a boxcar window size of 25 data points. 

Overall, the 24 mm OPL, and fused OPL data sets outperform the prediction of the 2 mm OPL data sets; this is an indication that the majority of the useful predictive bands come from the 24 mm data. A simple data fusion pre-processing step provides equivalent, or decreased prediction error in all tested cases for PLS regression with the exception of a boxcar window size of 10, which shows no change, or a slight increase in all cases. The 24 mm and fused OPL data sets perform within 5% of each other in all tested cases, but approximately 50% better than the 2 mm OPL set.

### 2.2. Ridge Regression

Ridge regression models returned a RMSEP range of 2.20–4.14 mEq O_2_/kg PV (Table 3). The optimized ridge regression models demonstrated comparable fits of the model to the calibration set and the test set. When performing ridge regression on the 2 mm OPL data set under no boxcar averaging conditions, bias is presented via 1 high influence point. This biased, high influence point also influences the fused OPLs data set. The influence of this point is significantly reduced in pathlength fused, boxcar averaged sets. However, under identical treatment conditions, the 24 mm OPL NIR regression shows significantly less, if any bias. Overall the best prediction results from the 24 mm OPL data with the 5 point boxcar window. Spectral fusion provides marginally decreased prediction errors when no boxcar averaging is employed; however, once any level of boxcar averaging is used, the 24 mm OPL outperforms the fused OPL. In all boxcar averaged data sets, spectral fusion is outperformed by the 24 mm OPL individual regressions, by a minimum of 10%. However, in the analysis of the raw spectral information, spectral fusion out performs the others by at least 3.5%. Interestingly, the raw spectral information (that is, no boxcar filtering) provides the most consistent Ridge prediction errors across the three data sets, with a range of 0.41 mEq O_2_/kg PV (no boxcar) compared to ranges of 1.94 mEq O_2_/kg PV (5 point boxcar), 1.74 mEq O_2_/kg PV (10 point boxcar), and 0.96 mEq O_2_/kg PV (25 point boxcar). Furthermore, the 2 mm OPL data set shows approximately a 20% decrease in prediction error when utilizing a 25 point boxcar window, while the 24 mm and fused OPL data sets show the same, or greater decrease in RMSEP values when utilizing a boxcar window of only 5 data points.

### 2.3. LASSO

LASSO regression shows prediction errors ranging from 1.80 to 3.90 mEq O_2_/kg PV (Table 4). All optimized LASSO models show comparable fits with the model to the calibration set and the test set. Most RMSEP were consistent for each OPL selection except for analyses employing the 2 mm OPL data. Considering overall performances, no definitive trend in combinations of OPL data and boxcar smoothing is apparent. The 24 mm OPL data set is the only method in which boxcar averaging shows a definitive increase in prediction error. However, the best LASSO models did demonstrate better RMSEP than the best PLS and ridge regression models, and comparable RMSEP with elastic net predictions.

### 2.4. Elastic Net

Elastic net regression shows prediction errors ranging from 1.91 to 3.83 mEq O_2_/kg PV (Table 5) with the exception of the raw, fused spectral information (no boxcar average pre-processing), and 5 point boxcar average pre-processing, fused spectral infomation. In the excluded cases, the elastic net regression prediction model fails, resulting in an RMSEP of 13.08 mEq O_2_/kg PV, and 12.11 mEq O_2_/kg PV and will be ignored for the immediate discussion of results. In the non-boxcar averaged data pre-processing trials, elastic net regression shows the smallest degree of bias of all 4 regression models tested. The elastic net regression model parameters leaned heavily to a LASSO model optimization with a slight contribution from a ridge regression model optimization in the penalty function. In many cases, the results of the elastic net and LASSO regressions produce almost identical RMSE values.

## 3. Discussion

It is noteworthy that the median RMSEP for the optimized models (Table 1) is only 2.06 mEq O_2_/kg PV given that the samples span 18 different classes of edible oils, multiple brands within each class, and occasionally multiple years within a brand. When considering all models, excluding the raw, and 5 point boxcar average fused elastic net, the models perform on average very similarly, with elastic net outperforming the others by a small margin, with RMSEP averages of 3.2 ± 0.94 mEq O_2_/kg PV for PLS, 3.2 ± 0.72 mEq O_2_/kg PV for ridge, 3.08 ± 0.85 mEq O_2_/kg PV for LASSO, and 2.99 ± 0.80 mEq O_2_/kg PV for elastic net. By comparison, many published studies present comparable RMSEP for studies employing only a single brand of one oil type. The relatively narrow spread of RMSEP across the 6 optimized models, +/−10% of the median mEq O_2_/kg PV, provides confidence that the best models are not spurious in nature—that is to say, one would lose confidence in a particular model were it to greatly outperform other models with similar structures and pre-processing protocols.

The regression methods investigated rely on different strategies to optimize the bias-variance trade-off. The methods that rely heavily on L-1 regularization (LASSO and elastic net) outperformed the models that rely heavily on L-2 optimizations (PLS and ridge). This is for both the 6 optimized models in Table 1 and as a trend across models for different combinations of OPL selection and boxcar smoothing. Elastic net does not, by default, rely heavily on L-1 regularization, but the λ-term in the optimized models showed that the best models were much closer to LASSO models then ridge models. The functional difference between LASSO and PLS/ridge models is that LASSO drives uniformative regression coefficients to zero. By contrast, PLS/ridge models minimize, but do not nullify, these coefficients. Hence, PLS/ridge leave a greater possibility for random errors to propagate through the calibration process. However, in some instances LASSO methods could eliminate needed variables, resulting in increased model bias. 

As elastic net does not rely solely on L-1 regularization, the regression coefficients do not reach 0, and are subsequently not removed from calculation as quickly, meaning elastic net can be more computationally taxing than ridge and LASSO. An example of this occurred in the raw and 5 point binned fused spectral information regression calculation, where no reasonable model could be achieved. For these specific cases, the large number of variables (spectral space) necessitated a limited set of tuning parameters. Therefore, what may have happened was a local minima was found by chance when the tuning parameters happened to align so that the local minima showed a lower apparent RMSE during tuning. However, the addition of a greater number of tuning variables could lead to extensive calculation time; beyond those feasible for realistic use. Interestingly in this study, the raw and 5 point boxcar, fused spectral information sets failed in all attempts, even when performed with parameters which performed well in other models.

The different performances of LASSO and PLS/ridge methods can be viewed through the implied distributions of true regression coefficients for the calibration problem. PLS/ridge assumes a normal distribution of regression coefficients while LASSO assumes a Laplace distribution [27]. While it is impossible to know the true distribution of regression coefficients with certainty for any calibration problem, in general LASSO tends to out-perform ridge regression when there are a few variables with significantly larger effects than many other variables [27]. While ridge is often superior when the data is comprised of many effects of equal importance across the observations [28]. One could imagine that, even with narrowing the region of interest to center on the vibrational modes, there are still many ‘baseline’ observations with minimal predictive ability in the employed data.

Although a LASSO model did provide the best RMSEP, multiple elastic net models nearly performed as well as the best LASSO model and the robustly consistent performance of elastic net across multiple OPL and boxcar window may make elastic net the preferred choice for NIR determination of PV in edible oils. The one instance where elastic net did not perform well was analyses of the fused data with no, or limited boxcar averaging. Theoretically, the fused data should perform comparable, at worse, to the best of the constituent data streams; however, the RMSEC, RMSECV and RMSEP were all significantly worse. This observation could stem from computational issues with the data set; many noisy variables in a wide, collinear matrix hinder the algorithm from approaching the true optimal solution. Analyses of the fused, no boxcar averaged, data by elastic net required extremely long calculation times, in the realm of 10 h. Applying a 10 point boxcar smoother greatly improved both the computation time and the model performance. 

**Figure 1:**
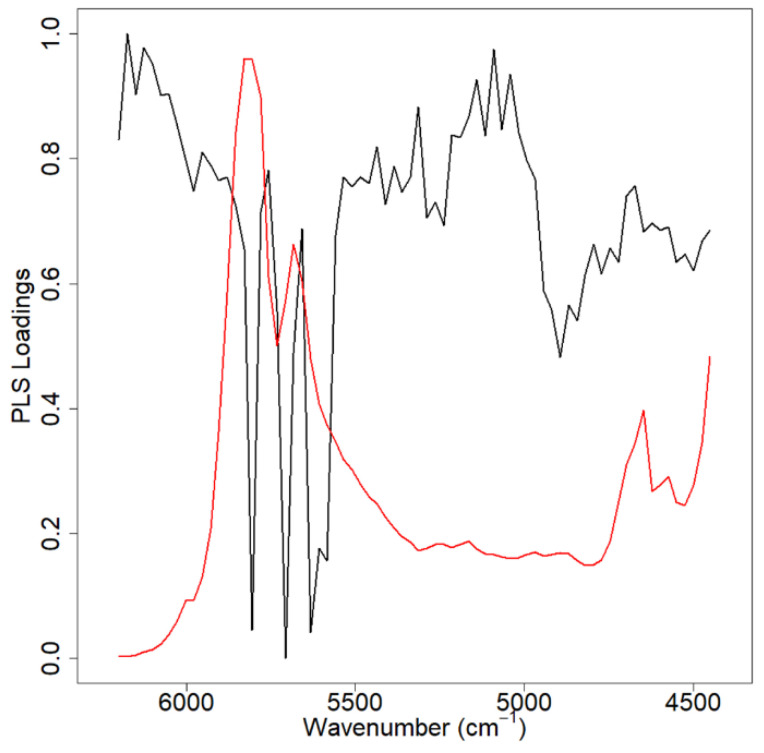

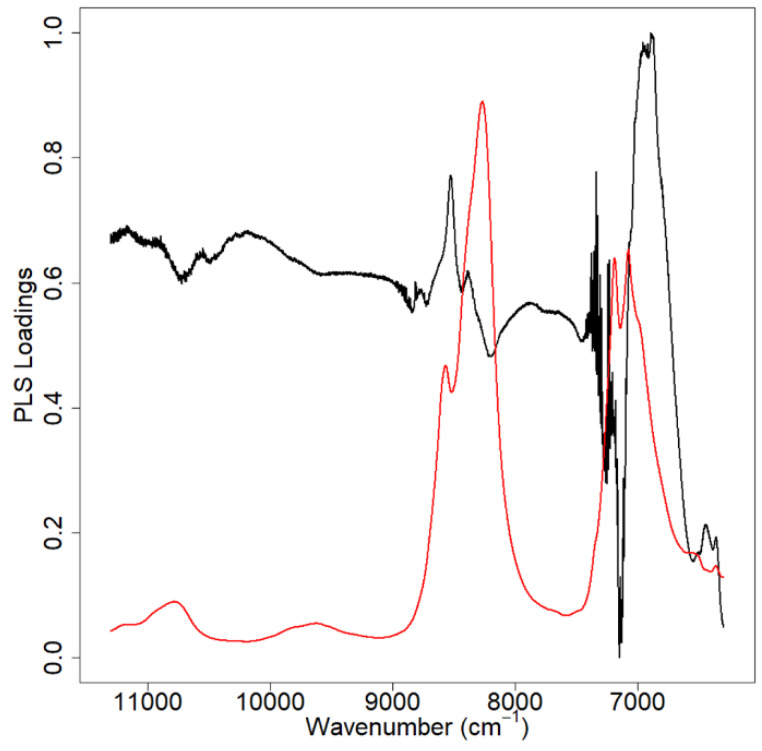
Normalized 2 mm OPL PLS loadings (top) and 24 mm OPL PLS loadings (bottom). In red is an example spectrum of the region of interest used in the regression analysis. In the 2 mm OPL loading (top), most prediction information comes from the 5500–6000 cm^−1^ features which is a CH 2nd overtone, while the 24 mm predictive information comes from the 6800–7200 cm^−1^ features (CH 1st overtone and R-OH 1st overtone) with added influence of the 8000–8800 cm^−1^ region (CH 2nd overtone).

Simple fusion of the two OPL data streams improved only the PLS model prediction performances. Furthermore, the fused PLS predictions do not significantly outperform the 24 mm OPL prediction performance. For the case of edible oils, a low wave number NIR spectral range of 4450–6200 cm^−1^ is elucidated from the 2 mm OPL cell, and a mid-spectral range of 6300–11300 cm^−1^ from the 24 mm OPL cell. The information in each region is unique to the OPL it was measured from and together can prove greater than the sum of the parts. However, as the spectral fusion analysis relies on the input of both input OPLs, poor-quality spectra collection, low spectral resolution, or high noise in either of the OPL spectra will greatly impact the performance of the final model. In this case, the 2 mm OPL spectral set derives its prediction information from small changes in a low-intensity spectral feature which are redundant to information captured in the 24 mm OPL, specifically the CH 2nd overtone (Figure 1) [24].

Boxcar averaging produces a minimal prediction improvement for all cases except the 24 mm OPL PLS and 24 mm OPL LASSO regressions. In general, the five-point and 10 point boxcar windows outperformed no boxcar averaging or using a 25 point boxcar window, although not unanimously. However, the 2 mm OPL unanimously performs best utilizing a large boxcar window. For the smaller boxcar windows, baseline noise reduction occurs in larger magnitude/proportion than information loss contained in that same spectrum. With a 25 point boxcar, vibrational bands begin to blur together, hindering the possibility to differentiate among PV-related changes in the spectra and other sources of variance such as oil type-related changes in the spectra. However, a 25 point boxcar window retains great enough spectral resolution to provide adequate regression predictions. Furthermore, the 2 mm OPL data set benefits from loss of spectral resolution as it helps to alleviate the spectral variation of the 2 mm OPL data set. Applying a minimal boxcar average to the data has the added advantage of greatly improving the computational time required to construct and optimize each model. Overall, the effect of spectral fusion and pre-processing demonstrates the preference of good data collection before complex statistical analysis.

## 4. Materials and Methods

### 4.1. Edible Oils

All edible oil samples were purchased from consumer supermarkets in the Newark Delaware region between 2012 and 2016. This multi-year range allowed for a wide range of PVs. All samples were stored at ambient conditions in the original container until aliquots of oil samples were collected for measurements. The starting set of oils consisted of 99 measured oil samples, with PVs ranging from1.7 to 80 mEq O_2_/kg, spanning 19 unique oil classes. Four outliers were identified and removed for a final 95 oil samples ranging 18 unique classes. Classes were assigned based upon manufacturer labeling, and therefore present the possibility of mislabeling, fraud or blend confusion. For example, oil blends of vegetable oil, vegetable and canola oil, and canola, sunflower and soybean oil were all classified uniquely even considering vegetable oil is most commonly a blend of soy and canola oils. Single measurements of both the AOCS iodometric titration oil PV and NIR spectrum were used for later analysis.

### 4.2. Peroxide Value Measurement

Peroxide values were determined using AOCS Cd 8-53 method by Eurofins Scientific inc., Nutrition Analysis Center, 2200 Rittenhouse Street, Suite 150 Des Moines, IA 50321. All edible oil PVs were measured close in time to the spectral analysis as possible to minimize additional aging the oil samples.

### 4.3. Spectroscopic Oil Measurement

**Figure 2:**
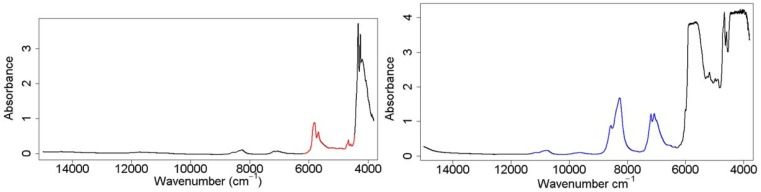
Raw collected spectra of individual OPLs, 2 mm OPL (**left**, red), 24 mm OPL (**right**, blue), with colored regions indicating the selected region used in prediction regression models. OPL fusion set is a direct combination of both highlighted regions into 1 data matrix.

Edible oil NIR spectra were also measured in the same time frame and are representative of the same oil PVs. Both the 2 and 24 mm OPL NIR spectroscopic measurements were performed at Lawrence Livermore National Laboratory using a Bruker Vertex 70 (Billerica, MA, USA) fitted with a room temperature InGaAs detector. The 24 mm NIR spectra were collected directly through the side wall of the glass scintillation storage vial, while the 2 mm OPL spectra were collected in a cuvette (Starna Cells, Inc. Atascadero, CA, USA, Spectrosil 1-Q-2). The recorded spectra were an average of 64 scans at 2 cm^−1^ resolutions, with a spectral range of 3799–14,998 cm^−1^ for the both OPL cells. All NIR spectra were referenced to an air blank, and no sample preparation was used. Both the exterior of the glass vial, and glass sample cell were wiped clean with a cloth before insertion in the instrument sample chamber. Usable region selection was dependent on two factors: all spectral features with an absorbance value greater than two were removed, and all regions of baseline with little variance were cut. For the 2 mm OPL, the region from 4450 to 6200 cm^−1^ was chosen; and for the 24 mm OPL, the region from 6300 to 11300 cm^−1^ was chosen (Figure 2).

### 4.4. Pre-Processing

Outlier removal was performed in two steps. First, two titrated samples were identified as statistical PV outliers, and thus removed. These had determined PV of 129.0 and 155.0 mEq O_2_/kg PV, well beyond the range of the other 97 samples and were identified through finding values outside of the range of 3 standard deviations from the mean in both directions. Two more of the titrated oil samples were identified as spectral outliers using Cook’s distance as an identifier. Cook’s distance was found by performing a PLS regression and predicting a linear fit to the regression. Any value outside of the range of 3 standard deviations from mean Cook’s distance were removed.

The oil samples were then randomly split into calibration and validation sets. The validation set was approximately 20% of the total samples, specifically 17 of 95 total samples, and contained proportional olive oil and non-olive oil samples, 8 olive oil and 9 non-olive oil. All samples were classified as specific oil type, as well a secondary classification of olive oil or non-olive oil. For blended oils: if the blend contained olive oil it was considered part of the olive oil class. The final, analyzed set contained 95 unique oil samples covering a range of 18 unique oil classes (Table 6).

All subsequent data pre-processing steps were performed individually on each set of data, with the pre-processing parameters determined from the calibration subset and subsequently applied to the validation subset. The two previously selected spectral regions were later combined to create a simple, fused data set used to perform the same, final regression model calculations. Fusing the different OPL measurements includes the unique aspects of each NIR spectra into the same regression, therefore, providing the greatest possible information, and therefore variance, to regress to. 

**Figure 3:**
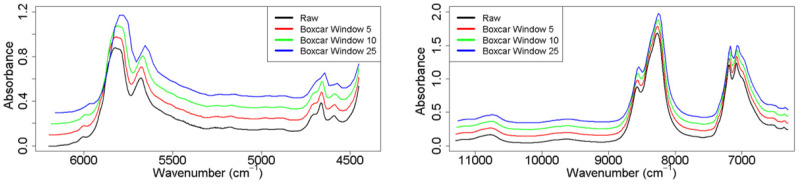
The 2 mm (**left**) and 24 mm (**right**) selected NIR regions under all boxcar averaging conditions. Only 1 example spectrum is shown, and the same oil sample was used for both example spectra sets. The spectral resolution loss at 25 point boxcar window size Both 5 point boxcar averaging (red spectrum) and 10 point boxcar averaging (green spectrum) do not loss a great enough amount of spectral resolution to affect prediction results significantly. 5 point (red), 10 point (green), and 25 point (blue) are offset by 0.1, 0.2 and 0.3 AU, respectively, for aesthetic purposes.

Each of the three data sets were tested using baseline correction techniques to determine which was most beneficial for creating regression prediction models for PV. Based on previous work, Ottaway et al. [24], 1st- and 2nd-order Savitzky–Golay smoothing was performed, with window sizes between 5 and 50; however, it showed no benefit to final prediction results. For these data sets, autoscaling provides adequate reduction in the random baseline variance within each spectra set. Small window spectral boxcar averaging is shown in previous work to further reduce the intrinsic noise in each observed data point when applied to systems with adequate spectral resolution [29]. Spectral boxcar averaging works to increase prediction efficiency by reducing the effect of random signal intensity changes, such as noise, while simultaneously increasing the effect of true chemical signals (Figure 3). Boxcar averaging spectral information has the added benefit of decreasing computational requirements and allowing simpler systems to perform the regressions require for proper PV prediction. 

### 4.5. Multivariate Analysis

PLS regression has been previously studied in depth to predict the PV of edible oil samples. The bilinear model for PLS regression is better able to address data sets with multicollinearity and data sets with more variables than predictors than univariate methods. Therefore, PLS is well suited for spectroscopy as the spectral space variables (X) are multicollinear while predictors (Y) are often only a scalar for each spectrum. The generalized model for PLS regression is the decomposition of the data (X) and predictor (Y) matrices to maximize the covariance of the X and Y scores matrices (T and U, respectively), with included error terms (E and F, respectively). This algorithmic method optimizes the regression coefficients used to create a least squares regression model.

(1) X = TP’ + E,

(2) Y = UQ’ + F,

To our knowledge ridge, LASSO and elastic net have not been used before to perform the same predictions on oil samples. Ridge, LASSO and elastic net are all based on the same extension on the Ordinary Least Squares (OLS) optimization equation [30]. The basic OLS optimization is modified using a penalty factor to bias the regression coefficients for model construction (Equations (3)–(5)).

All three regression techniques are the same expansion of the multilinear model approaching the regression coefficient penalization factor (k) in a different way. Ridge regression imposes a L-2 regularization penalty which varies the regression coefficient by a squared factor of the penalty value [31],

(3) $$\mathrm{Ridge}\;=\sum_{i=1}^{n}{\left(y_{i}-\sum_{j}X_{\mathrm{ij}}\beta_{j}\right)}^{2}+\;\lambda \sum_{j=1}^{p}\beta_{j}^{2},$$

where λ is equal to the tuning parameter and β is equal to the regression coefficients. This results in regression coefficients never reaching 0, meaning the model must account for more regression coefficients to properly calculate. LASSO regression prioritizes a simpler model and therefore uses a L-1 regularization to reduce some of the regression coefficients to 0, eliminating them from the calculation [32],

(4) $$\mathrm{LASSO}\;=\sum_{i=1}^{n}{\left(y_{i}-\sum_{j}X_{\mathrm{ij}}\beta_{j}\right)}^{2}\lambda \sum_{j=1}^{p}\left|\beta_{j}\right|,$$

where λ is equal to the tuning parameter and β is equal to the regression coefficients. This is performed by use an absolute value factor for the penalty factor. Elastic net combines both L-1 and L-2 regularization to maximize the benefits of both previous techniques [33],

(5) $$\mathrm{Elastic}\;\mathrm{Net}={\left|y-X\beta \right|}^{2}+\lambda [\alpha \sum_{j=1}^{p}\beta_{j}^{2}+\left(1-\alpha \right)\sum_{j=1}^{p}\left|\beta_{j}\right|],$$

where λ is equal to the tuning parameter and β is equal to the regression coefficients. In elastic net regression, the alpha parameter is a hyperparameter that acts as a decider between ridge and LASSO contribution with each extreme (1 or 0) being a pure regularization regression. However, this must be performed simultaneously, as sequential regularization of the data results in greater bias and overfitting, and therefore a worse prediction model than using either method individually. Ridge, LASSO and elastic net are chosen specifically for their enhanced ability to deal with high multicollinearity within data sets. All 3 OLS based optimization regression models approach the least squares regression model from the same algorithmic method, with related optimization. However, the optimization method for Ridge, LASSO, and elastic net varies greatly from the method used in PLS.

The root mean squared error of cross-validation (RMSECV) and percent variance explained from the PLS regression were used to determine the number of LVs for modeling. The RMSECV and root mean squared error of calibration (RMSEC) were found using the calibration set, then the validation set was projected into the model in order to calculate RMSEP as prediction error from the true measurements. Next, ridge, LASSO and elastic net regression were performed on each calibration set in two steps. The process of developing a prediction model was identical for ridge and LASSO, and only slightly varied for elastic net. First, for all three regression types, a separate tuning step was performed to identify the best fit parameter/s, hereby identified as lambda for ridge, and fraction for LASSO. The optimal lambda or fraction value was chosen via a plot of RMSE vs. log(lambda/fraction). For all calibration models, leave-one-out cross-validation was chosen as due to the limited number of samples. For elastic net regression, which varies both parameters simultaneously, the same method of tuning the parameters was used; however, the proper value was chosen from the parameter set, which provided the lowest RMSE vs. log(lambda and fraction) without obvious overfitting. An identical process was followed for the boxcar averaged data sets. In all cases, the RMSECV was calculated from leave-one-out cross-validation and the RMSEC was calculated as the calibration sets fit to the model. Lastly, the validation set was predicted into the regression model, and the RMSEP was calculated as a measurement from the true titration PVs.

### 4.6. Chemometric Analysis

All data analysis was performed using the packages, ‘doParallel’ [34], ‘R.matlab’ [35], ‘signal’ [36], ‘FactoMineR’ [37], ‘pls’ [38], and ‘caret’ [39] with R version 4.1.0 [40], with the package ‘elasticnet’ [41] used as a dependency inside the ‘caret’ package for calculating Elastic Net regressions.

## 5. Conclusions

Our results propose that, in the case of edible oil PV prediction, PLS regression presents reasonable, but substandard prediction results compared to other tested methods. Overall, elastic net regression provides the most consistent and, often, the most precise PV predictions when utilizing NIR spectroscopy, apart from some of the spectral fusion sets. The main benefit of elastic net regression is the inclusion of both the ridge and LASSO penalization parameter, allowing for a more precise estimation of the regression coefficient penalty factor. The result of considering two penalization parameters to produce prediction models creates models that are less affected by pre-processing method than the other presented models. While PLS, ridge, and LASSO individually provide reasonable predictions of PV, and can be improved though differing pre-processing methods, equivalent elastic net regression prediction results can be obtained without the need of these methods. Unfortunately, due to the more accurate prediction of regression penalty parameters, the same pre-processing methods have significantly less benefit than in other, simpler, regression methods. Furthermore, performing these regression predictions is not dependent on a small PV range, or different models for oil classes. This study shows that prior knowledge of the sample in question is not required for the accurate and precise prediction of PV. Most importantly, inclusion of varied oil classes and/or increased PV range, have little effect on PV prediction. This study provides excellent prediction of edible oil PVs, in agreement with previously published literature, without the need of class segregation or PV range limitation.

Dependent on the regression model in question, spectral fusion can provide significant benefit for PLS, moderate improvement for ridge, or seemingly no prediction improvement but possible prediction consistency improvement for LASSO and elastic net. However, the largest improvement of spectral fusion is the overall prediction consistency provided through the consideration of both OPLs simultaneously. The total prediction range of the spectral fusion sets, utilizing all regression models, is from 1.91 to 3.67 mEq O_2_/kg PV, a total range of 1.76 mEq O_2_/kg PV.

## Figures and Tables

**Table 1:** Figures of merit for the best-performing prediction models as well as highlighted influential or significant prediction regressions.

Regression Technique	Pre-Processing Method	Prediction Error (RMSEP) mEq O_2_/kg PV
PLS	25 point boxcar window, fused OPL	2.50
Ridge	5 point boxcar window, 24 mm OPL	2.20
LASSO	Raw spectra, 24 mm OPL	1.80
Elastic Net	10 point boxcar window, 24 mm OPL	1.91

**Table 2:** All RMSE values for PLS prediction regressions.

PLS		Raw	10 Point Binning	25 Point Binning	5 Point Binning
2 mm Pathlength	RMSECV	3.07	3.30	3.43	3.28
	RMSEC	2.32	2.45	2.54	2.47
	RMSEP	4.80	4.35	4.49	4.18
24 mm Pathlength	RMSECV	5.40	5.46	5.49	5.60
	RMSEC	3.61	3.62	3.62	3.63
	RMSEP	2.59	2.59	2.59	2.59
Fused Pathlength	RMSECV	4.14	4.12	4.14	4.11
	RMSEC	2.83	2.83	2.83	2.83
	RMSEP	2.54	2.54	2.64	2.50

**Table 3:** All RMSE values for ridge regression prediction regressions.

Ridge Regression		Raw	5 Point Binning	10 Point Binning	25 Point Binning
2 mm Pathlength	RMSECV	2.08	2.56	2.75	3.34
	RMSEC	1.49	1.8	1.94	2.08
	RMSEP	4.05	4.14	4.03	3.40
24 mm Pathlength	RMSECV	3.90	5.03	5.38	5.80
	RMSEC	3.80	2.80	3.05	3.23
	RMSEP	3.77	2.20	2.29	2.44
Fused Pathlength	RMSECV	2.93	3.26	3.56	3.61
	RMSEC	1.31	1.79	2.14	2.09
	RMSEP	3.64	2.81	2.99	2.66

**Table 4:** All RMSE values for LASSO prediction regressions.

Lasso		Raw	5 Point Binning	10 Point Binning	25 Point Binning
2 mm Pathlength	RMSECV	2.39	2.28	2.08	2.70
	RMSEC	1.01	1.33	1.45	1.26
	RMSEP	3.77	3.52	3.90	3.39
24 mm Pathlength	RMSECV	3.56	3.52	4.02	3.21
	RMSEC	1.72	1.89	1.58	1.65
	RMSEP	1.80	1.87	2.09	2.00
Fused Pathlength	RMSECV	2.36	2.34	2.25	2.29
	RMSEC	1.06	1.14	1.13	1.21
	RMSEP	3.64	3.78	3.67	3.48

**Table 5:** All RMSE values for elastic net prediction regressions.

Elastic Net		Raw	5 Point Binning	10 Point Binning	25 Point Binning
2 mm Pathlength	RMSECV	2.16	2.04	2.10	2.70
	RMSEC	1.22	1.01	1.31	1.26
	RMSEP	3.83	3.51	3.66	3.39
24 mm Pathlength	RMSECV	3.77	3.56	3.69	3.21
	RMSEC	1.48	2.06	2.19	1.65
	RMSEP	1.98	2.44	1.91	2.00
Fused Pathlength	RMSECV	6.05	7.22	2.25	2.29
	RMSEC	4.86	4.79	1.13	1.21
	RMSEP	13.08	12.11	3.67	3.48

**Table 6:** Class identification as indicated by oil manufacturer, and number of samples in each class. Classes with 0 samples indicate classes from previous work (Ottaway et al.) [24] with all samples removed as outliers, or not re-titrated in 2019.

Classes	Number of Samples	Samples in Validation Set	PV Range mEq O_2_/kg	Mean PV mEq O_2_/kg
Extra Virgin Olive Oil	26	5	12–50	26
Extra Light Olive Oil	6	1	25–40	31.8
Pure Olive Oil	8	0	16–44	31.6
Coconut Oil	0	0	-	-
Avocado Oil	2	1	14–33	23.5
Peanut Oil	5	0	27–56	41
Corn Oil	8	2	12–35	23
Grapeseed Oil	8	2	21–70	38.5
Safflower Oil	2	0	20–31	25.5
Hazelnut Oil	2	1	28–32	30
Flaxseed Oil	0	0	-	-
Almond Oil	5	0	26–45	36.6
Canola Oil	9	1	10–80	27.4
Avocado, Flaxseed, Olive oil	1	1	11	11
Sesame Oil	4	1	5.6–20	14.4
Blend of Canola and Vegetable	1	1	14	14
Vegetable	3	1	17–54	32.3
Blended Canola, Sunflower, Soybean	1	0	9.8	9.8
Sunflower	1	0	56	56
Walnut Oil	3	0	16–66	33.7

## Data Availability

The data presented in this study are available on request from the corresponding author.
